# Discovery of Bacterial Deaminases That Convert 5-Fluoroisocytosine Into 5-Fluorouracil

**DOI:** 10.3389/fmicb.2018.02375

**Published:** 2018-10-08

**Authors:** Agota Aučynaitė, Rasa Rutkienė, Daiva Tauraitė, Rolandas Meškys, Jaunius Urbonavičius

**Affiliations:** ^1^Institute of Biochemistry, Department of Molecular Microbiology and Biotechnology, Life Sciences Center, Vilnius University, Vilnius, Lithuania; ^2^Department of Chemistry and Bioengineering, Vilnius Gediminas Technical University, Vilnius, Lithuania

**Keywords:** metagenomics, deaminase, isocytosine, 5-fluorouracil, cancer therapy

## Abstract

Cytosine is one of the four letters of a standard genetic code, found both in DNA and in RNA. This heterocyclic base can be converted into uracil upon the action of the well-known cytosine deaminase. Isocytosine (2-aminouracil) is an isomer of cytosine, yet the enzymes that could convert it into uracil were previously mainly overlooked. In order to search for the isocytosine deaminases we used a selection strategy that is based on uracil auxotrophy and the metagenomic libraries, which provide a random pool of genes from uncultivated soil bacteria. Several genes that encode isocytosine deaminases were found and two respective recombinant proteins were purified. It was established that both novel deaminases do not use cytosine as a substrate. Instead, these enzymes are able to convert not only isocytosine into uracil, but also 5-fluoroisocytosine into 5-fluorouracil. Our findings suggest that novel isocytosine deaminases have a potential to be efficiently used in targeted cancer therapy instead of the classical cytosine deaminases. Use of isocytosine instead of cytosine would produce fewer side effects since deaminases produced by the commensal *E. coli* gut flora are ten times less efficient in degrading isocytosine than cytosine. In addition, there are no known homologs of isocytosine deaminases in human cells that would induce the toxicity when 5-fluoroisocytosine would be used as a prodrug.

## Introduction

Cytosine is a common cellular pyrimidine. It is a part of the cytidine triphosphate (CTP), which can serve as a high-energy molecule and a co-factor in metabolic processes. Cytosine is also one of the nitrogenous bases both in DNA and in RNA, which makes it an important molecule with many functions in the cell. It is a genomic “wild card” that is a key to generating genomic flexibility (Nabel et al., 2012). The cytosine modifications in messenger RNA precursors have significant impact on the functions of the proteins they encode (Gerber and Keller, 2001). Due to cytosine deamination, the AID/APOBEC family of cytidine deaminases have the unique potential to influence both epigenetic and genetic forms of heritable information, making these enzymes the link between both forms of inheritance (Chahwan et al., 2010). It is obvious that cytosine has objectives in the cell that are beyond the storage of information in the genome and most of these functions rely on the enzymes that are capable of modifying it.

Cytosine deaminase (CD) from *E. coli* is a member of the amidohydrolase superfamily that catalyzes the hydrolytic deamination of cytosine, forming uracil, and ammonia (Hall et al., 2011). Members of amidohydrolase superfamily are predominantly found in bacteria and also in certain fungi, but not in mammalian cells (Finn et al., 2016). Due to their ability to convert the non-toxic prodrug, 5-fluorocytosine (5-FC) into a well-known anticancer drug 5-fluorouracil (5-FU) (Raza et al., 2015) cytosine deaminases are among the key enzymes used in the prodrug-mediated control of variety of cancer types. Bacterial and yeast CDs have been investigated in the context of cancer therapy in detail by many research groups (Malekshah et al., 2016). After activation by CD, 5-FU can be further changed into potent pyrimidine antimetabolites by other cellular enzymes. It was shown that the 5-FU produced upon the action of this enzyme mediates the growth inhibition and apoptosis-mediated cell death of a variety of tumor types both *in vitro* and *in vivo* (Karjoo et al., 2016; Gaded and Anand, 2018). In this study, we applied our genetic screening system in order to search for new enzymes that could be beneficial for this type of cancer therapy. We chose isocytosine as the substrate for these putative enzymes.

**FIGURE 1:**
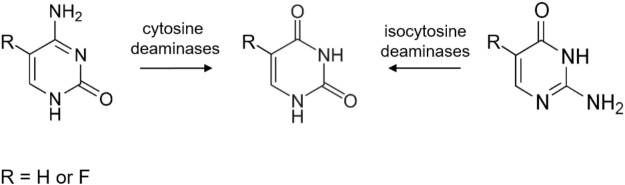
A schematic representation of the classical cytosine deaminase (CD) and the predicted isocytosine deaminase substrates and products.

Isocytosine (2-aminouracil) is an isomer of cytosine. Together with isoguanine it was one of the first non-natural nucleobases used in experiments of unnatural base pair studies. These studies seek to understand how the complementarity originally appeared in the forms of the A-T and G-C pairs and ultimately – to reveal the origin of the natural nucleic acids (Hirao et al., 2012). Isocytosine pairs in a normal manner with non-natural isoguanine and also in a “reversed Watson-Crick” manner with natural guanine. Therefore, it is used for structural studies of nucleic acids (Yang et al., 1998) as well as in physical-chemical studies involving metal complex binding (Gupta et al., 2004), hydrogen-bonding (Camacho-García et al., 2015), tautomerism and proton transfer (Delchev and Mikosch, 2007) in nucleobases. Moreover, following the logic of cytosine deaminase–5-fluorocytosine enzyme–prodrug pair, isocytosine could provide a base for a putative new prodrug 5-fluoroisocytosine, if it was used together with a putative isocytosine deaminase (**Figure 1**).

Metagenomes are an appealing pool of otherwise inaccessible genes that could reveal new enzymatic activities and even biochemical pathways (Popovic et al., 2015). In this study, we used the metagenomic libraries and an *E. coli* uracil auxotroph-based selection strategy in order to search for putative isocytosine deaminases. Since the expected enzymatic activity of the isocytosine deaminase is the conversion of isocytosine into uracil, we used an *E. coli* strain lacking several *pyr* genes that are responsible for the pyrimidine *de novo* biosynthesis (Aučynaitė et al., 2018). This strain is unable to grow in the defined synthetic medium without a source of uracil. Therefore, it could be used as a host strain for functional screening of the metagenomic libraries for the uracil generating enzymes. In this study isocytosine was used as a substrate supposing that the clones encoding the appropriate deaminases would convert isocytosine to uracil hence allowing the growth on the mineral medium.

## Materials and Methods

### Bacterial Strains, Plasmids, Primers, Media and Reagents

*Escherichia coli* DH5α (Thermo Fisher Scientific) was used for routine DNA manipulations. *E. coli* DH10B (Thermo Fisher Scientific) was used for disruption of *pyr* genes to obtain the DH10BΔ*pyr* strain (Aučynaitė et al., 2018). *E. coli* BL21(DE-3; Novagen) was used to produce the recombinant deaminase proteins.

High copy number cloning vector pUC19 (Thermo Fisher Scientific) was used for the preparation of metagenomic libraries (Aučynaitė et al., 2018). Vectors for inducible expression of C-terminally 6xHis-tagged proteins pET21b(+), pET28a(+; Novagen) and pQE-70 (Qiagen) were used for cloning of the URA3, Vcz, and *E. coli* CD CodA genes, respectively.

Standard techniques were used for DNA manipulations (Sambrook and Russell, 2001). The URA3 deaminase gene was amplified by PCR using primers *URA3FW* 5′-ATATACATATGGCCAAAACACTCTTGG-3′ and *URA3RV* 5′-TATATCTCGAGTTCACCCATGACC-3′, digested with *Nde*I and *Xho*I and cloned into the corresponding site of pET21b(+) vector. The Vcz deaminase gene was amplified by PCR using primers *VczFW* 5′-ATATACCATGGACAAAAGAACGCTGC-3′ and *VczRV* 5′-TATATCTCGAGCAGCCGATGCCGGTT-3′, digested with *Nco*I and *Xho*I and cloned into the corresponding site of pET28a(+) vector. The *E. coli* CD CodA gene was amplified by PCR using primers *CodAFW*: 5′-ATTAAGCATGCCGAATAACGCTTTA-3′ and *CodARV*: 5′-TAATTAAGCTTTCAACGTTTGTAATCGAT-3′, digested with *Sph*I and *Hind*III and cloned into the corresponding site of pQE-70 vector. All of the DNA primers were synthesized at Metabion International AG. All of the restriction endonucleases were purchased from Thermo Fisher Scientific.

*Escherichia coli* strains transformed with recombinant plasmids were grown in nutrient broth (NB; Oxoid, Thermo Fisher Scientific) medium supplemented with either 100 mg/L ampicillin or 50 mg/L kanamycin, as required, at 37°C with aeration (unless noted otherwise). *E. coli* DH10BΔ*pyr* cells transformed with metagenomic libraries were grown in M9 minimal medium with casamino acids (Ausubel et al., 2003) supplemented with 100 mg/L ampicillin, 15 mg/L kanamycin, 20 mg/L cytosine, isocytosine or uracil, as required, at 37°C with aeration. A final concentration of 0.1 mM IPTG was added to the M9 minimal medium for induction of *E. coli* CD CodA gene expression.

Uracil and 5-fluorouracil were purchased from Sigma-Aldrich Co., Isocytosine was purchased from Combi-Blocks Inc. Cytosine was purchased from Alfa Aesar. 5-fluoroisocytosine was synthesized using the established protocols, with slight modifications (Chen et al., 2009 WO/158011 A1).

### Over-Expression and Purification of the Recombinant 6xhis Tagged Isocytosine Deaminase Proteins

Vcz and URA3 deaminase genes were cloned into pET28a(+) or pET21b(+) vectors, respectively, and transformed in to the BL21(DE-3) cells. The resulting bacteria were grown in LB medium containing 50 mg/L kanamycin (for pET28a(+)) or 100 mg/L ampicillin (for pET21b(+)). The culture was grown at 37°C until OD_600_ reached 0.5–0.6. It was then cooled on ice and the inducer isopropyl-1-thio-β-D-galactopyranoside (IPTG, Thermo Fisher Scientific) was added to a final concentration of 0.5 mM. The induced cells then were incubated at 20°C overnight. The cells were then collected by centrifugation, resuspended in 50 mM TRIS–HCl, pH 8, and disrupted by sonication at 750 W for 1 min using a VC-750 ultrasound processor (Sonics 0026 Materials, Inc.). Cell debris was removed by centrifugation at 16000 × *g* for 10 min.

Cell extracts were loaded onto a Ni-NTA column (GE Healthcare) previously equilibrated with 50 mM TRIS–HCl, pH 8. The adsorbed proteins were eluted with 50 mM TRIS–HCl, pH 8 using linear gradient of 0–500 mM imidazole. The fractions containing the proteins were pooled and desalted by dialysis against 50 mM TRIS–HCl, pH 8. The purity of recombinant proteins was confirmed by electrophoresis on a 12% SDS–PAGE gel visualized by Coomassie Brilliant Blue staining. The concentration of recombinant proteins was measured using Lowry method (Lowry et al., 1951) with bovine serum albumin as the standard.

### Substrate Specificity Assays

The enzymatic reactions were carried out at 37°C in 50 mM TRIS–HCl, pH 8.0 buffer and incubated for 1 h. The 20 μL final volume of the reaction mixture contained 1 μg of purified recombinant 6xHis tagged Vcz or URA3 deaminase protein and 20 mM of substrate (cytosine, isocytosine, or 5-fluoroisocytosine).

After the incubation, 1 μL of the reaction mixture was used for the thin layer chromatography (TLC) analysis. TLC was performed on aluminum sheets coated with silica gel 60 F254 using the methanol:chloroform (1:9) mixture as a mobile phase. The spots were detected and visualized under the 254 nm UV light.

HPLC-MS analyses were performed using a high performance liquid chromatography system, equipped with a photo diode array detector (SPD-M20A) and a mass spectrometer (LCMS-2020), equipped with an electrospray ionization (ESI) source. The chromatographic separation was conducted using a YMC Pack Pro column, 3 mm × 150 mm at 40°C and a mobile phase that consisted of 0.1% formic acid water solution (solvent A) and acetonitrile (solvent B). Mass spectrometry data was acquired in both positive and negative ionization mode and analyzed using the LabSolutions LCMS software.

### Enzyme Activity Measurements

The activity of isocytosine deaminases were assayed spectrophotometrically at 21°C by monitoring the decrease in absorbance at 285 nm (ε_285_ = 3760 M^-1^ cm^-1^) or 260 nm (ε_260_ = 2715 M^-1^ cm^-1^) resulting from the deamination of isocytosine or 5-fluorisocytosine to uracil or 5-fluorisocytosine, respectively, by using a Helios gamma UV-visible spectrophotometer (Thermo Fisher Scientific). The reaction was initiated by the addition of the appropriate amount of the enzyme to 0.05 M TRIS–HC1, pH 8.0, buffer supplemented with isocytosine (5–700 μM) and the initial reaction rates were recorded. The kinetic parameters (*k*_cat_ and *K*_m_) of the purified Vcz and URA3 deaminases were determined by fitting the experimental data from three independent experiments using a Michaelis-Menten equation (SigmaPlot 10).

### Synthesis of 5-Fluoroisocytosine

Ethylfluoroacetate (1.93 mL, 20 mmol) and ethylformate (1.74 mL, 20 mmol) were added to the cooled suspension of sodium hydride (960 mg, 20 mmol) in 30 mL of anhydrous ether. The reaction mixture was stirred for 20 h at room temperature. The solvent was evaporated and the residue was cooled in an ice bath. Guanidine hydrochloride (1.38 g, 60 mmol) was neutralized by addition to a solution of sodium ethylate (2.5 M in ethanol, 24 mL) cooled in an ice bath. The solution was stirred for 30 min, the formed sodium chloride was filtered and the filtrate was added to the cooled reaction mixture. The mixture was refluxed for 17 h. After the reaction was completed (as monitored by TLC), the formed brownish precipitate was filtered. The filtrate was concentrated under reduced pressure and acidified with 1 N HCl solution to pH 4.0. The crude reaction mixture was twice purified by reverse phase column chromatography (C-18 cartridges, water/methanol mixture, 10:0→10:2). The solvents were removed under reduced pressure to afford 780 mg (30% yield) of 5-fluoroisocytosine. MS (ESI^+^), m/z 128.05 (M-H)^-^, 130.05 (M + H)^+^. UV λ_max_ 263 nm. ^1^H NMR (DMSO–d_6_, 400 MHz): δ = 6.50 (s, 2H, NH_2_); 7.64 (d, 1 H, *J* = 4.3 Hz, CH); 11.42 (s, 1H, NH). ^13^C NMR (DMSO–d_6_, 100 MHz): δ = 142.43; 144.77; 153.25; 156.15.

## Results

### Screening for Isocytosine Deaminases

**FIGURE 2:**
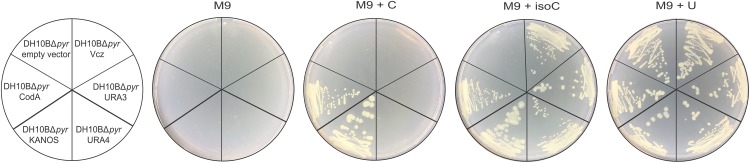
*In vivo* functionality of *E. coli* CD (CodA, in pQE70 expression vector) and the DNA fragments that were selected from four metagenomic libraries: KANOS, URA4, URA3, and Vcz (in pUC19 vector). Empty pQE70 vector was used as a negative control. Minimal medium (M9) was supplemented 100 mg/L ampicillin, 15 mg/L kanamycin, 0.1 mM IPTG, and either with 20 mg/L cytosine (M9 + C), isocytosine (M9 + isoC) or uracil (M9 + U, positive control).

The *E. coli* DH10BΔ*pyr* strain is unable to grow in the defined synthetic medium without a source of uracil, because its pyrimidine *de novo* biosynthesis pathway has been disrupted. Therefore, these cells can be used as a host while searching for isocytosine deaminases in the metagenomic libraries, if the M9 minimal medium is supplemented with isocytosine as a sole source of uracil. Of the several positive hits, three transformants exhibited strong growth on isocytosine, but not cytosine (**Figure 2**, Vcz, URA3, and URA4); one, which could use isocytosine as well as cytosine, was also selected as control (**Figure 2**, KANOS), as it was reminiscent of classical CD (**Figure 2**, CodA). It is shown in **Figure 2** that proteins encoded all four of the selected DNA fragments are functional *in vivo* and are able to convert isocytosine into uracil thus restoring the growth phenotype of *E. coli* DH10BΔ*pyr* cells.

Plasmid DNA was isolated from these transformants and sequenced using primer-walking method. The presence of a single ORF encoding a deaminase was revealed in each of the DNA fragments. The DNA fragments, ORFs and corresponding proteins were named after the metagenomic libraries they were found in: Vcz, URA3, URA4, and KANOS [accession numbers MH015236, MH015234, MH015235, and MH015237 in GenBank (Benson et al., 2012), respectively].

**FIGURE 3:**
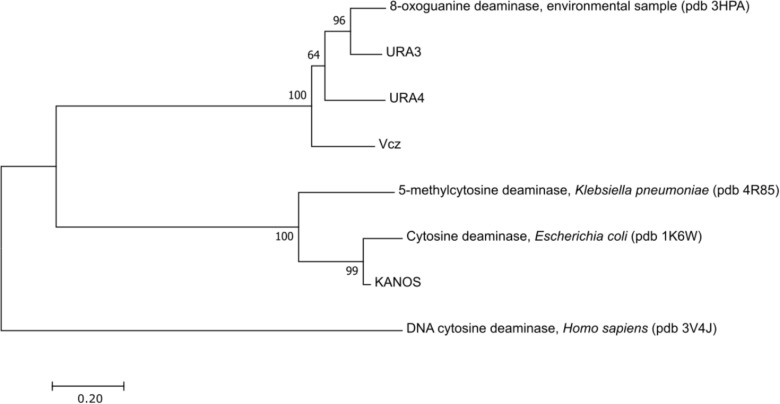
Evolutionary relationships of deaminases. The evolutionary history was inferred using the Neighbor-Joining method (Saitou and Nei, 1987). The optimal tree with the sum of branch length = 3.9 is shown. The percentage of replicate trees in which the associated taxa clustered together in the bootstrap test (1000 replicates) are shown next to the branches (Felsenstein, 1985). The tree is drawn to scale, with branch lengths in the same units as those of the evolutionary distances used to infer the phylogenetic tree. The evolutionary distances were computed using the Poisson correction method (Zuckerkandl and Pauling, 1965) and are in the units of the number of amino acid substitutions per site. The analysis involved 8 amino acid sequences. All positions containing gaps and missing data were eliminated. There were a total of 207 positions in the final dataset. Evolutionary analyses were conducted in MEGA7 (Kumar et al., 2016). This analysis demonstrates that the enzymes found in metagenomic libraries belong to two groups as depicted in the tree: the classical cytosine deaminases (KANOS) and the 8-oxoguanine deaminases (URA3, URA4, and Vcz).

**FIGURE 4:**
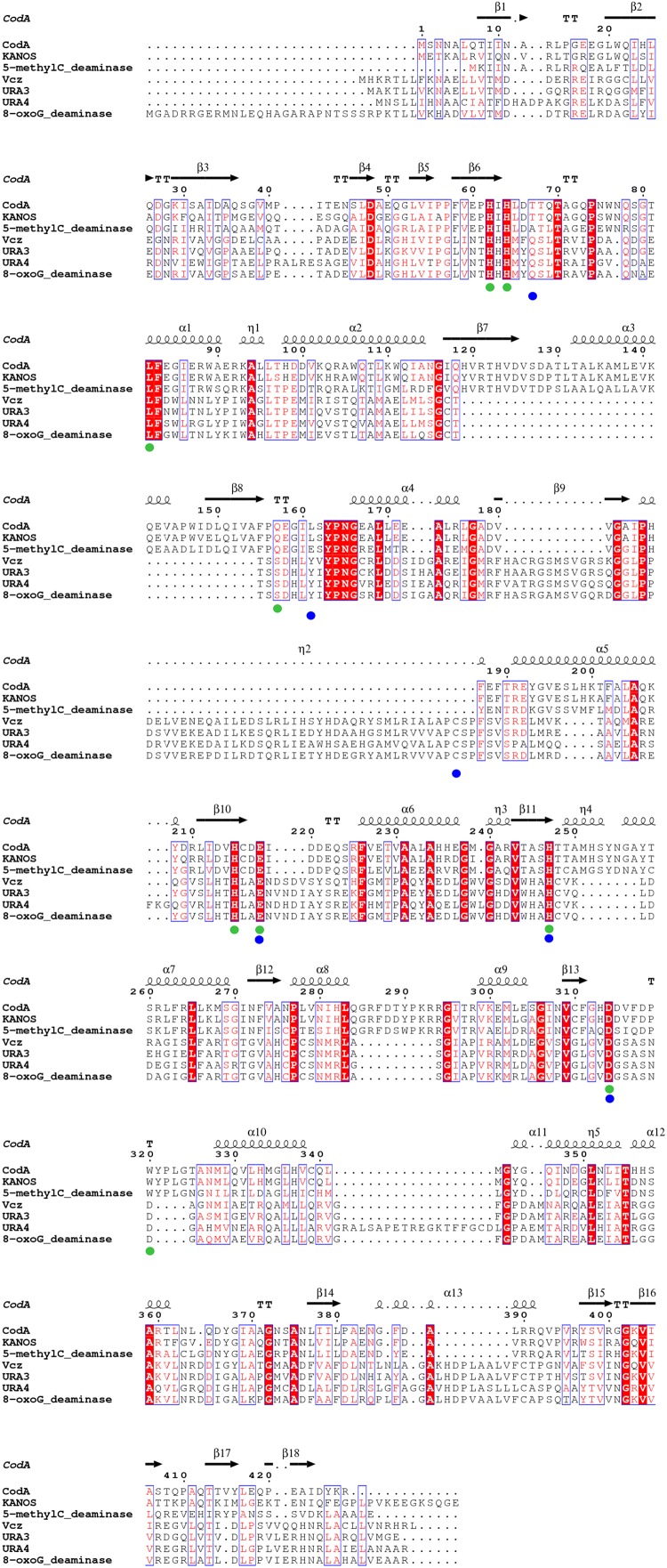
Multiple amino acid sequence alignment of deaminases from the metagenomic libraries (KANOS, Vcz, URA3, and URA4) and those with confirmed functions and tertiary structures. CodA: *E. coli* cytosine deaminase, amino acid residues forming its active site are indicated in green circles (Ireton et al., 2002); 5-methylC_deaminase: *Klebsiella pneumoniae* 5-methylcytosine deaminase (Hitchcock et al., 2014); 8-oxoG_deaminase: 8-oxoguanine deaminase, amino acid residues forming its active site are indicated in blue circles (Hall et al., 2010). Highly similar residues are in red and framed in blue, strictly identical residues are in white on a red background. The alignment was performed using Clustal Omega (Sievers et al., 2011) and ESPript (Robert and Gouet, 2014).

Ensuing phylogenetic analysis using MEGA7 (Kumar et al., 2016) demonstrated that these proteins belong to two phylogenetic groups as depicted in **Figure 3** (human single-strand DNA CD APOBEC3G (Li et al., 2012) was used as an outgroup). The KANOS protein is closely related to the classical cytosine deaminases (represented by CodA of *E. coli* (Ireton et al., 2002), see also **Figure 4**), therefore it was not chosen for further experiments. The other three proteins are closely related to 8-oxoguanine deaminases represented by the 8-oxoguanine deaminase from an environmental sample of Sargasso sea (Hall et al., 2010), but are different from a bacterial 5-methylcytosine deaminase (Hitchcock et al., 2014), which is related to the cytosine deaminases.

### Enzymatic Activity of the Recombinant Deaminase Proteins

**FIGURE 5:**
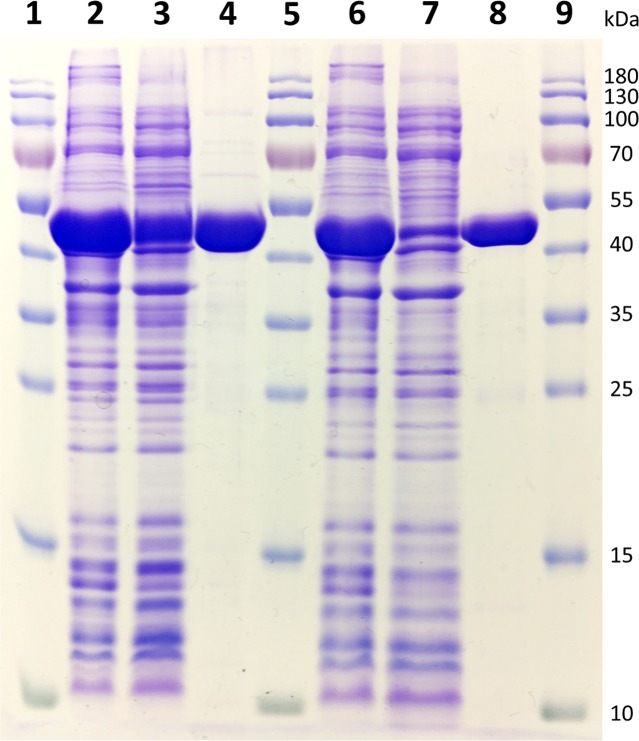
12% SDS–PAGE gel representing the purification of recombinant Vcz and URA3 deaminases. 1, 5, and 9: PageRuler^TM^ Prestained Protein Ladder (Thermo Fisher Scientific); 2: total proteins obtained from induced *E. coli* BL21(DE-3) bacteria transformed with pET28a-Vcz; 3: soluble protein fraction of *E. coli* BL21(DE-3) bacteria transformed with pET28a-Vcz; 4: ∼40 μg of recombinant 6xHis-tagged Vcz deaminase; 6: total proteins obtained from induced *E. coli* BL21(DE-3) bacteria transformed with pET21b-URA3; 7: soluble protein fraction of *E. coli* BL21(DE-3) bacteria transformed with pET21b-URA3; 4: ∼30 μg of recombinant 6xHis-tagged URA3 deaminase.

**FIGURE 6:**
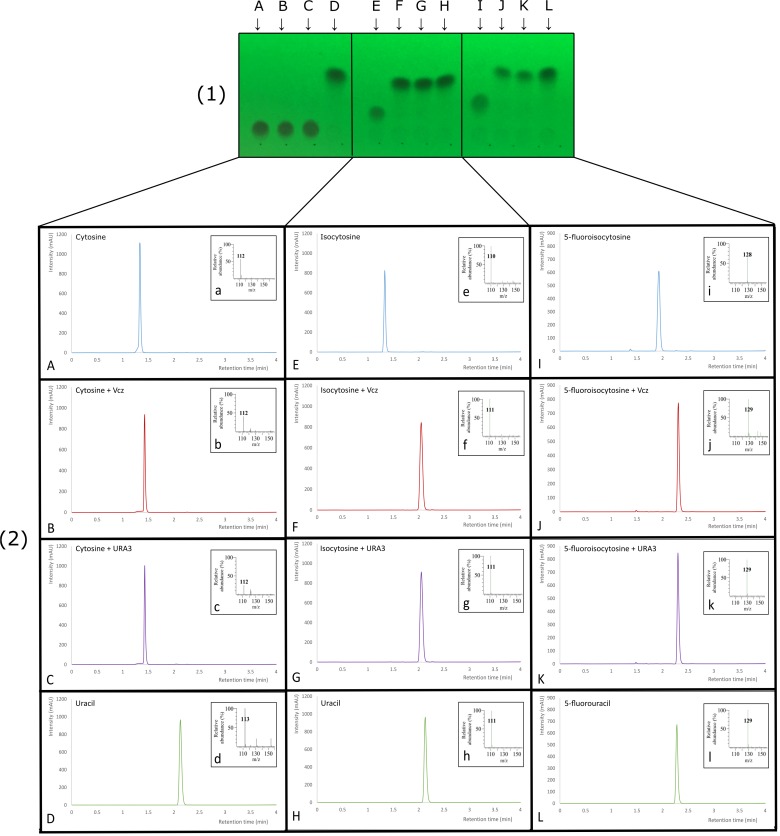
Thin layer chromatography plate **(1)** and corresponding LC-MS analyses **(2)** of substrates **(A,E,I)**, enzymatic isocytosine deaminase Vcz **(B,F,J)** and URA3 **(C,G,K)** reactions and products **(D,H,L)**. **(A)** TLC and UV chromatogram of cytosine standard, **(a)** MS-spectra (*m/z* of 112 consistent with [M + H]^+^ of cytosine), **(B)** TLC and UV chromatogram of the reaction mixture using cytosine and Vcz deaminase, **(b)** MS-spectra (*m/z* of 112 consistent with [M + H]^+^ of cytosine), **(C)** TLC and UV chromatogram of the reaction mixture using cytosine and URA3 deaminase, **(c)** MS-spectra (*m/z* of 112 consistent with [M + H]^+^ of cytosine), **(D)** TLC and UV chromatogram of uracil standard, **(d)** MS-spectra (*m/z* of 113 consistent with [M + H]^+^ of uracil), **(E)** TLC and UV chromatogram of isocytosine standard, **(e)** MS-spectra (*m/z* of 110 consistent with [M-H]^-^ of isocytosine), **(F)** TLC and UV chromatogram of the reaction mixture using isocytosine and Vcz deaminase, **(f)** MS-spectra (*m/z* of 111 consistent with [M-H]^-^ of uracil), **(G)** TLC and UV chromatogram of the reaction mixture using isocytosine and URA3 deaminase, **(g)** MS-spectra (*m/z* of 111 consistent with [M-H]^-^ of uracil), **(H)** TLC and UV chromatogram of uracil standard, **(h)** MS-spectra (*m/z* of 111 consistent with [M-H]^-^ of uracil), **(I)** TLC and UV chromatogram of 5-fluoroisocytosine standard, **(i)** MS-spectra (*m/z* of 128 consistent with [M-H]^-^ of 5-fluoroisocytosine), **(J)** TLC and UV chromatogram of the reaction mixture using 5-fluoroisocytosine and Vcz deaminase, **(j)** MS-spectra (*m/z* of 129 consistent with [M-H]^-^ of 5- fluorouracil), **(K)** TLC and UV chromatogram of the reaction mixture using 5-fluoroisocytosine and URA3 deaminase, **(k)** MS-spectra (*m/z* of 129 consistent with [M-H]^-^ of 5- fluorouracil), **(L)** TLC and UV chromatogram of 5-fluorouracil standard, **(l)** MS-spectra (*m/z* of 129 consistent with [M-H]^-^ of 5- fluorouracil).

Two of the deaminase proteins (URA3 and Vcz) were overproduced in the *E. coli* BL21(DE-3) cells and purified using the Ni-ion chromatography (the purification steps are shown in **Figure 5**). The molecular weights of URA3 and Vcz (approx. 50 kDa) corresponded to the theoretical ones (48.8 and 49.5 kDa, respectively) (**Figure 5**). Third putative deaminase URA4 was not purified since the attempts to clone it into the expression vectors have failed. The recombinant URA3 and Vcz deaminases were tested for the enzymatic activity *in vitro* by incubating them with isocytosine and other substrates. As depicted in **Figure 6**, neither of these deaminases use cytosine as a substrate (**Figures 6A–D**), but they do convert isocytosine into uracil (**Figures 6E–H**) as well as 5-fluoroisocytosine into 5-fluorouracil (**Figures 6I–L**). The Vcz and URA3 enzymes efficiently catalyzed the deamination of isocytosine but were not active toward cytosine. For Vcz, the values of *k*_cat_, *K*_m_, and *k*_cat_/*K*_m_ for isocytosine were 1.64 ± 0.06 s^-1^, 1860 ± 45 μM, and 8.8 ± 0.3 × 10^2^ M^-1^ s^-1^, respectively. For URA3, the values of *k*_cat_, *K*_m_, and *k*_cat_/*K*_m_ for isocytosine were 2.00 ± 0.15 s^-1^, 1085 ± 120 μM, and 1.8 ± 0.1 × 10^3^ M^-1^ s^-1^, respectively. For Vcz, the values of *k*_cat_, *K*_m_, and *k*_cat_/*K*_m_ for 5-fluorisocytosine were 0.1 ± 0.007 s^-1^, 1270 ± 128 μM, and 7.9 ± 0.08 × 10^1^ M^-1^ s^-1^, respectively. For URA3, the values of *k*_cat_, *K*_m_, and *k*_cat_/*K*_m_ for isocytosine were 0.02 ± 0.003 s^-1^, 1330 ± 305 μM, and 1.5 ± 0.3 × 10^1^ M^-1^ s^-1^, respectively.

## Discussion

The isocytosine deaminases that were discovered in this study are all members of the amidohydrolase superfamily of enzymes that catalyze the hydrolysis of a wide range of substrates and contains most of the enzymes known to catalyze the deamination of nucleobases. In all known cases, the nucleophilic water molecule is activated through complexation with a mononuclear or binuclear metal center (Zn^2+^ in the case of 8-oxoguanine deaminases) that is perched at the C-terminal end of the β-barrel core within a (βα)_8_ structural domain (Seibert and Raushel, 2005). The phylogenetic analysis that includes deaminases of amidohydrolase superfamily with known enzymatic activities and tertiary structure solved, demonstrated that the deaminases we have discovered fall into 2 distinct groups. The first group includes the URA4, URA3, and Vcz, which are closely related to 8-oxoguanine deaminases – these enzymes have previously been shown to act on isocytosine (Hall et al., 2010). The second group includes the classical CD (CodA from *E. coli*), 5-methylcytosine deaminase Kpn00632 from *Klebsiella pneumoniae* (Hitchcock et al., 2014) and KANOS (**Figure 3**). Based on the similarity to 8-oxoguanine deaminase discovered by Hall et al. (2010), we tested the enzymatic activity of both URA3 and Vcz enzymes and discovered that they are capable of converting both guanine into xanthine and 8-oxoguanine into uric acid. The limited solubility of guanine and 8-oxoguanine prevented accurate detection via TLC. Nonetheless, according to the HPLC-MS results that were obtained, URA3 and Vcz deaminases convert guanine into xanthine and 8-oxoguanine into uric acid, although the detection of UV spectra and molecular ions is complicated (data not shown). The sequence comparison of newly discovered isocytosine deaminases with the ones that act on either cytosine, 5-methylcytosine or 8-oxoguanine (**Figure 4**) allowed us to make predictions regarding the requirements for the enzymatic activity and substrate specificity. The tertiary structures and the enzymatic activities of the above mentioned deaminases were studied in detail (Ireton et al., 2002; Hall et al., 2010, 2011; Hitchcock et al., 2014). Sequence alignment (**Figure 4**) demonstrates that all these deaminases are metal proteins with binuclear metal centers that are defined by the conservative His62, His64, His215, and Asp314 residues (numbering according to CodA in **Figure 4**, green dots). These residues were previously reported as involved in metal coordination and substrate binding in the active site of *E. coli* CD (Ireton et al., 2002). The residues that form the active center of 8-oxoguanine deaminases (Hall et al., 2010) are conserved in the Vcz, URA3 and URA4 deaminases (**Figure 4**, blue dots). Three of these residues are conservative in CodA (and in 5-methylcytosine deaminase as well) and are involved in its active site formation (**Figure 4**, CodA numbering: Glu218, His247, and Asp314, green and blue dots). However, the Asp315, which is present in CodA and KANOS (CodA numbering in **Figure 4**), is replaced by glycine in URA3, URA4, Vcz, and 8-oxoguanine deaminase (or by serine in 5-methylcytosine deaminase). This residue is critical for the substrate specificity of deaminases: CodA is essentially unable to catalyze the deamination of 5-methylcytosine, but when the Asp315 residue was replaced by alanine, the mutant CodA enzyme displayed activity toward 5-methylcytosine (Hitchcock et al., 2014). Comparison of 3D structures of CodA and the 8-oxoguanine deaminase demonstrates that β7/α3/β8 helixes are missing in the latter as well as in the Vcz, URA3, and URA4 deaminases, but an α3_10_ (η2) helix is present instead, in which a cysteine residue that is involved in active site formation is present (**Figure 4**, η2 helix, blue dot). In contrast to all known isocytosine/cytosine deaminases, URA4 protein contains an additional loop between helixes α10 and α11. The functional role of this structural element is not clear. It was demonstrated that 8-oxoguanine deaminase from *Pseudomonas aeruginosa* PA01 is capable of isocytosine deamination, albeit with 10-fold lower efficiency than that of 8-oxoguanine. Moreover, cytosine was not a substrate of this protein (Hall et al., 2010). The URA3 and Vcz deaminase values of *k*_cat_/*K*_m_ for isocytosine were similar to those obtained by Hall et al. (2010), using the 8-oxoguanine deaminase from *Pseudomonas* sp. However, their homolog from Sargasso sea metagenome is a tenfold more efficient in deamination of isocytosine, as well as is the *E. coli* CD (Hall et al., 2011).

Although the isocytosine deaminases, as predicted, do convert isocytosine into uracil, another substrate – 5-fluoroisocytosine – is a gateway to a potential future application of newly discovered enzymes, namely, the enzyme-prodrug therapy of cancer. The purpose of cancer therapy is to kill cancer cells selectively without harming the non-cancer cells. The classical bacterial or yeast CD and its prodrug 5-fluorocytosine (5-FC) have been one of the most investigated enzyme-prodrug pairs for many years (Malekshah et al., 2016). This type of cancer therapy relies on the non-toxic prodrug 5-FC, which is converted to its active form, 5-fluorouracil (5-FU), by CD activity (Mullen et al., 1992; Huber et al., 1993).

The 5-FU, which is a common chemotherapy drug, is further converted into potent pyrimidine antimetabolites by other cellular enzymes and can inhibit thymidylate synthase which leads to the cell cycle arrest and apoptosis (Yata et al., 2012). 5-FU is widely used in the treatment of a range of cancers, including that of the aero-digestive tract, breast, head, and neck, but the greatest response rates were observed in the case of colorectal cancer. However, the administration of 5-FU is toxic to the patients – up to 80% of administered 5-FU is broken down in the liver (Longley et al., 2003). Hence, the efficient tumor-localized expression of CD enzymes remains one of the main problems that the enzyme-prodrug therapy has to face.

The use of the non-toxic prodrug and localized expression of CD enables the reduction of the systemic side effects of 5-FU. Still, it remains one of the problems with CD/5-FC enzyme/prodrug therapy, mainly because of the presence of CD in normal gut flora, which is also able to convert 5-FC to 5-FU (Harris et al., 1986; Malet-Martino et al., 1991). We propose that 5-fluoroisocytosine, together with the new URA3 or Vcz isocytosine deaminases, could be used as a novel enzyme-prodrug pair. We believe that the gut flora would not metabolize 5-fluoroisocytosine as efficiently as 5-fluorocytosine. Although it was shown that the CD from *E. coli* is able to catalyze the deamination of isocytosine, the kinetic parameters (*k*_cat_/*K*_m_) differ by a 10-fold in favor of cytosine (Hall et al., 2011). Moreover, there are no known homologs of Vcz and URA3 isocytosine deaminases in the human cells, as revealed by BLAST (Altschul et al., 1990) analysis (data not shown). The new isocytosine deaminase/5-fluoroisocytosine enzyme/prodrug pair would alleviate the toxic side effects of cytosine deaminase/5-fluorocytosine pair in cancer therapy. More biochemical experiments may be required in order to determine the exact kinetic parameters of these enzymes with different substrates and also to pinpoint the exact amino acids involved in the catalysis. The alanine scanning and/or random mutagenesis experiments similar to those performed for the 5-FC deaminase (Mahan et al., 2004a,b) would allow the selection of the most efficient variants of URA3 and Vcz enzymes. In order to test the applicability of isocytosine deaminase/5-fluoroisocytosine enzyme/prodrug pair for cancer gene therapy, several experiments are under way. These additional studies are aimed at production of cell lines/bacterial strains that express the isocytosine deaminases and also at the action of this novel enzyme/prodrug pair on cancer cells *in vitro* and in animals.

## Conclusion

Three isocytosine/8-oxoguanine deaminases that belong to the amidohydrolase superfamily were discovered using soil-based metagenomic libraries, a selection method employing *E. coli* uracil auxotrophy and isocytosine as a substrate. It was demonstrated *in vitro* that two of these deaminases are capable of converting isocytosine, but not cytosine, into uracil as well as 5-fluoroisocytosine into 5-fluorouracil. The latter finding suggests that these enzymes, together with 5-fluoroisocytosine, could be used for cancer therapy as a novel enzyme/prodrug pair, which might alleviate problems associated with the classical cytosine deaminase/5-fluorocytosine enzyme/prodrug pair.

## Author Contributions

AA performed the genetic screens, cloning, enzymatic activity measurements, and wrote the manuscript. RR purified the recombinant proteins and reviewed the manuscript. DT synthesized 5-fluoroisocytosine, performed the HPLC-MS analyses, and reviewed the manuscript. RM planned the experiments, analyzed the results, provided the metagenomic libraries, and reviewed the manuscript. JU planned the experiments, analyzed the results, and wrote the manuscript.

## Conflict of Interest Statement

AA, RR, DT, RM, and JU declare potential financial interests in the future development and commercialization of the isocytosine deaminases. Vilnius University has filed a Lithuanian patent application (LT2017 533).
